# Autophagy takes it all – autophagy inducers target immune aging

**DOI:** 10.1242/dmm.049345

**Published:** 2022-01-31

**Authors:** Heidi Zinecker, Anna Katharina Simon

**Affiliations:** 1 Turkish-German University, Department of Molecular Biotechnology, 34820, Beykoz/Istanbul, Turkey; 2 NDORMS, The Kennedy Institute of Rheumatology, University of Oxford, Oxford OX3 7FY, UK

**Keywords:** Aging, Autophagy, Autophagy inducer, Immune system

## Abstract

Autophagy, as the key nutrient recycling pathway, enables eukaryotic cells to adapt to surging cellular stress during aging and, thereby, delays age-associated deterioration. Autophagic flux declines with age and, in turn, decreases in autophagy contribute to the aging process itself and promote senescence. Here, we outline how autophagy regulates immune aging and discuss autophagy-inducing interventions that target senescent immune cells, which are major drivers of systemic aging. We examine how cutting-edge technologies, such as single-cell omics methods hold the promise to capture the complexity of molecular and cellular phenotypes associated with aging, driving the development of suitable putative biomarkers and clinical bioassays. Finally, we debate the urgency to initiate large-scale human clinical trials. We give special preference to small molecule probes and to dietary interventions that can extend healthy lifespan and are affordable for most of the world's population.

## Abstract (precis)

**Summary:** Aging of the immune system drives ineffective immune responses in older people. Autophagy-inducing dietary interventions and drugs rejuvenate adaptive immune cells and could extend a healthy lifespan or offer new strategies to treat age-related diseases.

## Introduction

By 2050, it is estimated that 2 billion people, i.e. >20% of the total world population, will be over 60 years old (key facts on ageing and health, WHO, 2021). The high proportion of older people poses a challenge and burden for health systems around the world. The shared aetiology of age-associated disorders, such as atherosclerosis, cardiovascular disease, osteoarthritis, osteoporosis, type 2 diabetes, cancer and neurodegenerative diseases is still poorly understood (Nikolich-Žugich, 2018). However, a growing body of evidence suggests that aging of the immune system significantly contributes to multimorbidity and mortality of older adults. Overall, older adults are not only prone to age-related conditions but are also more susceptible to microbial infections and they exhibit diminished vaccine responses due to decreased immune function (Haynes, 2020; Mueller et al., 2020).

In this Perspective, we specifically focus on the systemic deterioration of immune function with age, which is accompanied by inflammaging, a chronic state of innate immune activation causing systemic low-grade inflammation (Furman et al., 2019). Immune aging, characterized by dysregulated and impaired immune function, entails changes to immune cell number, function, and maturation, as well as transcriptional and metabolic reprogramming in individual cells. It is the result of thymic involution with reduced T cell output and reduced B cell production in the bone marrow, leading to a decrease in peripheral naïve adaptive immune cells. The aged immune system shows hallmark features, including impaired immune reactions to new antigens and perturbed memory responses. This has been best studied in T cell immunity, which is key to the adaptive immune response (Goronzy and Weyand, 2019). With age, an increasing number of less-effective memory T cell subsets acquire senescence-like features (Aiello et al., 2019; Zhang et al., 2016). Furthermore, T cells can be a contributing factor to inflammatory cytokine production, and T cells from old mice proliferate less (Desdín-Micó et al., 2020).

“A growing body of evidence suggests that aging of the immune system significantly contributes to multimorbidity and mortality of older adults”

Age-related cellular changes can manifest as either senescence – with its defining cell-cycle arrest, or as cellular aging, which is characterised by damaged macromolecules and organelles (Campisi and di Fagagna, 2007; López-Otín et al., 2013). Senescence occurs through stress-induced damage, leading to cell-cycle arrest coupled with a senescence-associated secretory phenotype (SASP) – a cellular state shown to be beneficial in embryonic development, tissue repair and regeneration (Demaria et al., 2014; Muñoz-Espín et al., 2013; Storer et al., 2013).

It is also known that senescent cells shut down division but do not undergo apoptosis and, in this way, prevent the development of cancer (Serrano et al., 1997). On the one hand, the chronic presence of senescent cells is harmful to surrounding cells and tissues owing to their secretion of cytokines, chemokines, proteases and bioactive lipids, and the attraction of inflammatory cells – all of which are thought to contribute to inflammaging (Gorgoulis et al., 2019; Zhou et al., 2021). On the other hand, cellular aging is a consequence of the accumulation of damaged macromolecules (DNA, proteins and lipids) and organelles (e.g. mitochondria) and occurs in long-lived, post-mitotic cells (Campisi and di Fagagna, 2007; López-Otín et al., 2013). The combination of persistent environmental insults, and the decline of repair and removal mechanisms leads to this increased damage (Desdín-Micó et al., 2020; Goronzy and Weyand, 2019).

Autophagy is a candidate pathway known to prevent both cellular aging and chronic low-grade inflammation (Klionsky et al., 2021). This evolutionary conserved process degrades and removes damaged cell material, such as protein aggregates and organelles, to maintain cellular and tissue homeostasis. It enables adaptation to cellular stress, including excessive reactive oxygen species (ROS) production and metabolic stress, such as nutrient starvation. The core process of autophagy is instigated by inhibition of mechanistic target of rapamycin complex 1 (mTORC1) and/or activation of 5′ AMP-activated protein kinase (AMPK), both of which are canonical inducers of autophagy in response to metabolic stress. The core machinery and main pathways that sustain autophagy are thoroughly defined in various research papers and reviews (Dikic and Elazar, 2018). In summary, cytoplasmic material is entrapped in double-membrane autophagosomes that then fuse with lysosomes in which their content is degraded and recycled. The three types of autophagy can be classified as macroautophagy, microautophagy and chaperone-mediated autophagy. Macroautophagy, generally referred to as autophagy, is mostly a highly selective, receptor-mediated pathway (selective autophagy) that targets, degrades and recycles specific cargo. This pathway enables adaptation to environmental challenges through the removal of pathogens or damaged organelles. In contrast, nonselective autophagy occurs in response to starvation, providing energy and new anabolic substrates.

“Autophagic flux declines with age; decreases in autophagy, however, contribute to senescence and the aging process itself”

Unfortunately, autophagic flux declines with age; decreases in autophagy, however, contribute to senescence and the aging process itself (Aman et al., 2021; Rubinsztein et al., 2011). Therefore, defects in autophagy are connected to a variety of human illnesses, including immune-associated and age-related diseases, which are both often linked to increased inflammation (Aman et al., 2021; Klionsky et al., 2021).

## Autophagy shapes adaptive immunity

Recently, a plethora of studies revealed that selective autophagy, in close association with immunometabolism, is key in modulating immunity and immune cell dynamics (Germic et al., 2019; Metur and Klionsky, 2021). In addition, autophagy is further involved in differentiation and proliferation of immune cells, although what exactly underlies molecular mechanisms remains partly elusive (Clarke and Simon, 2019). Mounting evidence indicates that mitophagy, which encompasses selective degradation of damaged or excessive mitochondria, is an especially crucial regulator of innate immune cell function (Gkikas et al., 2018). Autophagy also prevents mitochondrial DNA escaping into the cytoplasm by maintaining mitochondrial homeostasis, which inhibits initiation of type I interferon signalling and, ultimately, inflammation (Riley and Tait, 2020). Furthermore, autophagy is essential for T cell immunity and its decline with age leads to immunosenescence (Phadwal et al., 2012; Puleston and Simon, 2014).

Drug discovery has identified numerous small compounds that can reverse age-associated effects via autophagy

**Fig. 1.**
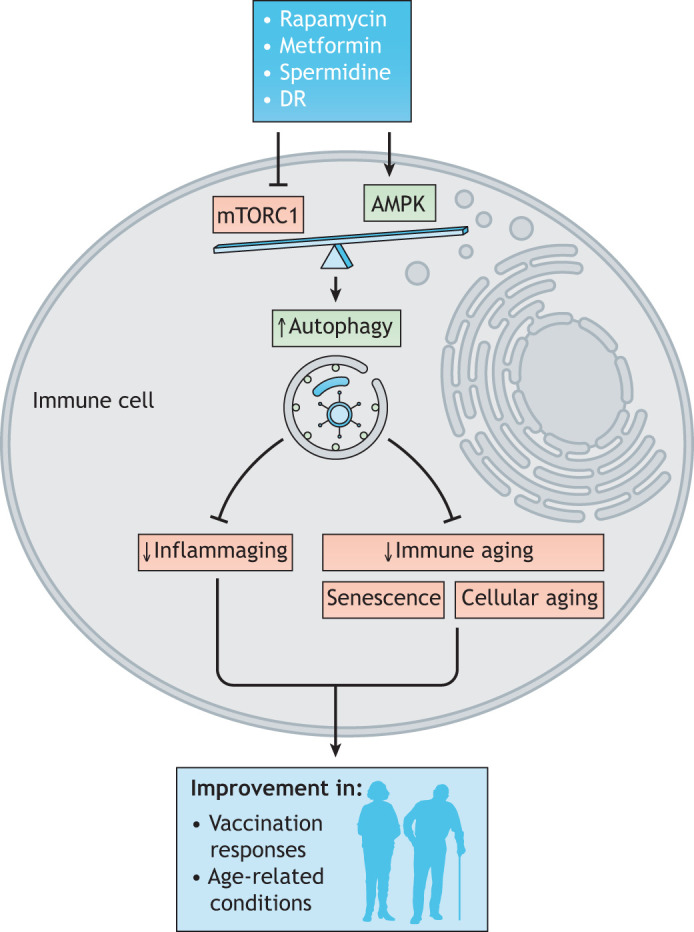
Rejuvenation of immune cells by autophagy-inducing drugs, such as rapamycin, metformin and spermidine, and dietary interventions (DR).

During the last two decades, several trailblazing studies have suggested that innate and adaptive immunity are key to fight not only infectious diseases but also non-communicable diseases including typical age-related conditions, such as cancer. Considering that autophagy facilitates adaptive immune cell activation (Clarke and Simon, 2019; Hubbard et al., 2010; Pua et al., 2007) and differentiation (Mortensen et al., 2011; Riffelmacher et al., 2017), and partially reverses systemic immunosenescence via modulating T cell immunity, clinical implementation of autophagy inducers provides high therapeutic potential. Drug discovery has identified numerous small compounds that can reverse age-associated effects via autophagy. These have been suggested to extend median and maximal lifespan, underpinned by *in vivo* and *in vitro* data obtained in various animal models (recently reviewed by Aman et al., 2021). Importantly, lifestyle and nutrition, particularly exercise (He et al., 2012) and dietary restriction (Hansen et al., 2018; Scott et al., 2004; Ulgherait et al., 2021) enhance the autophagy pathway. Several repurposed and already FDA-approved drugs that either inhibit mTORC1 or activate AMPK have recently gained considerable attention as promising immunoprotective interventions that could be translated into clinic within the next decade (Fig. 1). Here, we focus on the three most-promising drugs and on dietary restriction as a lifestyle change.

## Autophagy induction by rapamycin

The natural macrolide compound rapamycin was first isolated from the soil bacterium *Streptomyces hygroscopicus* found on Easter Island in 1972. Rapamycin and its derivatives known as rapalogs, such as everolimus and temsirolimus, are highly potent allosteric inhibitors of mechanistic Target of rapamycin (mTOR). The protein kinase mTOR is formed by two complexes: mTORC1 and mTORC2. mTORC1 predominantly controls nutrient-sensitive growth signalling and is a master regulator of fundamental homeostatic processes, such as autophagy and translation. mTORC2 mainly modulates cell proliferation and the actin cytoskeleton, and has been shown to regulate chaperone-mediated autophagy via lysosomal localisation (Arias et al., 2015). In addition, mTORC2 may negatively modulate autophagy via signalling pathways distinct from mTORC1, although this is only partly understood (Ballesteros-Álvarez and Andersen, 2021).

Rapamycin (also known as sirolimus) is approved by the FDA as an immunosuppressive agent to prophylactically treat liver transplant rejection. The other rapalogs are also widely used in cancer therapy. They have been extensively characterized over the last 30 years and are, to date, among the most-promising anti-aging compounds owing to their significant effect on lifespan in yeast, nematodes, fruit flies and mice, even when given later in life (Bjedov et al., 2010; Harrison et al., 2009; Selvarani et al., 2021). Furthermore, it was shown that rapamycin can delay the onset of age-associated diseases in mammals, including a decline in immune function (Flynn et al., 2013; Kaeberlein and Galvan, 2019). Here, we only focus on rapamycin's effect on the immune system, including its effect on responses to viral infections and vaccination, and its ability to – at low doses – prevent cellular senescence (Araki et al., 2009).

Numerous studies in mice were conducted to test *in vivo* efficacy of rapamycin (Selvarani et al., 2021). Hematopoietic stem cells (HSCs) are multipotent precursors with the capacity to both self-renew and differentiate into functional immune cells, red blood cells and platelets. Yet, HSC functions significantly decline with age. Rapamycin treatment restored self-renewal and expansion of HSCs, and enabled efficient vaccination against a lethal challenge with influenza virus in aged mice (Chen et al., 2009). Araki et al., among others, showed that inhibition of mTORC1 activity by rapamycin results in enhanced generation of memory CD8+ T cells providing long-term immunity, which is key in promoting vaccine efficiency, but whether this occurred via autophagy has not been tested (Araki et al., 2009; Turner et al., 2011). Moreover, other studies in mice demonstrated that low doses of rapamycin boost cross-strain protection against infection with lethal influenza A virus by modifying the antibody repertoire and immunoglobulin class-switching following vaccination (Keating et al., 2013). Ground-breaking results from a phase II clinical trial with 264 human participants revealed a significant effect of RAD001 (Everolimus) administration on vaccine efficacy (Mannick et al., 2018). Participants in the study who had received a daily low dose of RAD001 (100 or 500 μg) for 6 weeks exhibited a significantly stronger response to flu vaccine and had fewer infections in the following year (Mannick et al., 2018). Whether the effect is modulated via autophagy is unknown. However, long-term administration of rapamycin *in vivo* results in detrimental side effects, i.e. on glucose tolerance mediated by inhibition of mTORC2 (Lamming et al., 2012). Thus, highly specific mTORC1 inhibitors are needed to avoid adverse effects. Recently, the *in vitro* and *in vivo* application of a novel potent rapalog that selectively targets mTORC1, revealed that many negative side effects could be prevented (Schreiber et al., 2019).

In addition, a recent drug screen using human primary fibroblasts identified rapamycin as a potent inhibitor of SASP (Herranz et al., 2015). As previously discussed, SASP and senescence in immune cells as well as in HSCs, contribute to age-associated pathologies (Chen et al., 2009; Desdín-Micó et al., 2020). Therefore, preventing immune senescence in older people with rapamycin or other rapalogs appears to be a promising approach in the development of geroprotective treatments for diseases that disproportionally affect the elderly, such as seasonal flu, COVID-19 and osteoarthritis (Bischof et al., 2021; Dhanabalan et al., 2021 preprint).

## Metformin – more than an antidiabetic drug

Metformin has been used as an antidiabetic drug for 60 years. However, in the 1940s, it was originally utilized as an anti-influenza and anti-malaria drug that dampens inflammation. Although its many targets and various off-target effects are a drawback, it generally is a safe drug with a low toxicity profile. A systematic review of studies in human subjects reveals the geroprotective potential of metformin (Campbell et al., 2017). Diabetes patients taking metformin suffer from fewer age-related pathologies and have reduced mortality rates compared to diabetes patients who received non-metformin treatment and even to healthy subjects. Intriguingly, these effects are apparently independent of the effect of metformin on diabetes (Campbell et al., 2017).

Several studies implicate metformin as a modulator of immune functions, including differentiation of innate and adaptive immune cells and anti-inflammatory effects through activation of AMPK via phosphorylation and subsequent inhibition of mTORC1 (Barzilai et al., 2016; Martin-Montalvo et al., 2013). Furthermore, metformin inhibits mitochondrial complex 1, a key enzyme complex in the electron transport chain, which subsequently leads to an increased AMP:ATP ratio and to activation of AMPK (El-Mir et al., 2000; Foretz et al., 2014; Owen et al., 2000). In addition, inhibition of mitochondrial complex 1 reduces mitochondrial ROS production, oxidative stress and, consequently, senescence, as well as secretion of proinflammatory cytokines, such as IL-6 and IL-1b (Schuiveling et al., 2018; Soberanes et al., 2019). In line with metformin's effects on the mitochondria and immune differentiation, it was recently shown that it improves the memory immune response in order to contain *Mycobacterium tuberculosis* infection in mice by metabolically reprograming memory CD8+ T cells (Böhme et al., 2020). CD8+ T cells treated with metformin exhibited transcriptional changes, and increased mitochondrial health and cell survival (Böhme et al., 2020).

Interestingly, recent evidence shows that metformin activates autophagy in primary CD4+ T cells of older individuals, i.e. aged 57–68 years (Bharath et al., 2020). Owing to reduced autophagy, aging CD4+ T cells show a STAT3-mediated Th17 inflammatory profile that is characterized by elevated production of interleukins, which contribute to inflammation and senescence. Metformin reverses this Th17 phenotype toward a juvenile CD4+ T cell phenotype by enhancing autophagy and mitochondrial bioenergetics, but not mitophagy (Bharath et al., 2020).

Currently, metformin is gaining huge attention as a repurposed drug, as analyses of clinical data revealed reduced mortality rates of COVID-19 patients who had been treated with the anti-diabetic drug (Crouse et al., 2021; Ibrahim et al., 2021; Varghese et al., 2021). These findings suggest an urgent need of large clinical trials with metformin as prophylactic intervention for immune aging and age-associated diseases.

## Spermidine – ‘all good things come in threes’

Spermidine is another autophagy-inducing compound with promising anti-aging potential (Eisenberg et al., 2009). This natural occurring polyamine is a metabolic product of the arginine-metabolism of eukaryotic cells and the human microbiota. Several food products, including aged cheese, fermented soy, wheat germs, nuts, peas and mushrooms, contain high quantities of spermidine. Polyamine-rich foods boost longevity in yeast, worms, flies (Eisenberg et al., 2009) and mice (Eisenberg et al., 2016), and prevent age-induced memory impairment in mice (Schroeder et al., 2021). Recently, prospective clinical data manifested a link between longevity and spermidine. Concomitantly, spermidine levels are decreased in aged somatic cells, including in *Drosophila* brain and immune cells, such as mature lymphocytes (Gupta et al., 2013; Puleston et al., 2014). Analogous to the aforementioned autophagy-inducing drugs, spermidine also modulates activation and function of immune cells (Gupta et al., 2013; Puleston et al., 2014).

Importantly, supplementation of spermidine in old mice leads to induction of autophagy in memory T and B cells, and rejuvenates B cell responses in an autophagy-dependent manner (Zhang et al., 2019). Interestingly, spermidine can act as a donor for hypusination of the translation factor eIF5A, which is integral to its activation and enables efficient biosynthesis of the autophagy transcription factor TFEB (Zhang et al., 2019). In addition, spermidine can promote differentiation of CD4+ T cells towards a regulatory Foxp3+ phenotype with an anti-inflammatory profile, which was abrogated in *Atg5*-deficient T cells that are incapable of autophagy initiation (Carriche et al., 2021). The generation of a memory CD8+ T cell pool is key to mediate protective immunity against microbial infections (Goronzy and Weyand, 2019). Spermidine only enhances the production of virus-specific memory CD8+ T cells when these cells are autophagy proficient (Puleston et al., 2014; Zhang et al., 2016). Intriguingly, the decline of autophagy and immune function in T cells of old humans (mean age 77.6 ± 6.6 years) can be restored by spermidine supplement (Alsaleh et al., 2020). Currently, human clinical trials are being initiated to examine whether spermidine promotes an efficient immune response to vaccines in elderly subjects. Recently conducted preclinical and clinical trials revealed safety and tolerability of daily spermidine supplementation (Schwarz et al., 2020; Wirth et al., 2019). Launching long-term therapeutic trials might prove its usage to rejuvenate aged immune cells.

## Dietary restriction

The cheapest autophagy-inducing drug is probably lifestyle intervention, including dietary restriction (DR). DR comprises a variety of feeding regimens, including the reduction in nutrients without malnutrition or omitting specific parts of the diet, such as carbohydrates, and a variety of fasting methods, including intermittent and periodic fasting, that are – in theory – easily implementable (Buono and Longo, 2019; Green et al., 2021; Longo et al., 2021). Very recently, several seminal papers revealed a close relationship between DR, inflammation and immune cell function and dynamics in several organisms, including humans (Collins et al., 2019; Jordan et al., 2019; Ma et al., 2020; Nagai et al., 2019). Nagai and colleagues, investigated the effect of fasting and refeeding on gut immunity in mice. Peyer's patches are composed of lymphoid follicles that act as intestinal immune sensors. In response to temporary fasting, lymphocytes, particularly naïve B cells, circulate between Peyer's patches and the bone marrow (Nagai et al., 2019). The physiological relevance of this dynamic behaviour is unclear but demonstrated that fasting regulates gut immunity. Another study elucidated the effect of reduced food intake on circulating monocytes, which orchestrate induction and maintenance of inflammation (Jordan et al., 2019). Fasting reduced the pool of peripheral monocytes and diminished their inflammatory activity without compromising a proper immune response against microbial infections (Jordan et al., 2019). In a comprehensive single-cell RNA-sequencing approach, Ma and colleagues analysed the effects of both aging and caloric restriction by creating a single-cell transcriptome atlas, generated from 210,000 cells and nuclei of *Rattus norvegicus*, which allowed the dissection of alterations at a cellular and molecular level (Ma et al., 2020). The data show that a variety of immune cells, such as neutrophils and natural killer T cells, are strongly affected by DR in a tissue-dependent manner. This allowed the authors to identify age-related gene expression patterns and signalling pathways that are reversed by DR, thereby showing that inflammation is the most-important factor in the aging process. Moreover, DR reverses inflammaging – a ground-breaking discovery (Ma et al., 2020). Importantly, fasting regimens inhibit the AKT/mTOR pathway, thereby inducing autophagy (Fontana and Partridge, 2015; Nagai et al., 2019). In response to DR, mTOR signalling is differentially regulated in memory T cells, and these cells also increasingly migrate and accumulate in the bone marrow niche. Of note, this upregulated memory T cell homing has been associated with an enhanced memory T cell response to a second bacterial challenge (Collins et al., 2019).

In *C. elegans*, DR activates autophagy through transcriptional changes and has been suggested to be necessary, but not sufficient, to promote longevity (Hansen et al., 2008). Despite accumulating research in this area, further mechanistic studies that establish a causal link between autophagy and extended life span are needed (Madeo et al., 2015).

## Tackling the aging crisis – future perspectives

Immune aging, as a predominant risk factor for various chronic age-related diseases, has been suggested to accelerate the course of the organism's aging process. Autophagy-inducing agents reverse immune aging, senescence and SASP phenotypes, and rejuvenate the immune system. Of the drugs and interventions mentioned, those that inhibit mTOR (rapamycin, DR) also downregulate protein synthesis and, therefore, may display unwanted effects (Nofal et al., 2017; Zhao et al., 2015). More specific autophagy modulators are, therefore, needed.

The majority of those findings was assessed in mice and other model organisms. To enable effective human studies, we suggest the development and monitoring of suitable biomarkers, and parameters that capture immune senescence and cellular aging, in addition to immune-response biomarkers. Blood sampling in humans is feasible and easy to manage. Thus, a plethora of blood senescence markers can simultaneously be monitored when utilizing advanced flow cytometry, including senescence-associated beta-galactosidase (SA β-gal), CDKN1A (p21) and TP53 (p53), as well as markers of mitochondrial and lysosomal health, autophagic flux, and inflammation, including IL6, IL8, IL1B and TNFA (Zhou et al., 2021). In addition, novel biomarkers should be validated and implemented. Several such candidates, including growth differentiation factor 15 (GDF15) (Tanaka et al., 2018), matrix metalloproteinase-1 (MMP1), stanniocalcin-1 (STC1), and serine protease inhibitors (SERPINs) have been discovered by proteomic analysis (Basisty et al., 2020). In parallel to large-scale clinical trials, novel technologies, such as spatial and single-cell transcriptomic analysis in combination with machine-learning algorithms, will help to identify molecular targets of the drugs, and expression patterns that are associated with senescence and immune aging in differing immune cell populations (Mogilenko et al., 2021).

The current COVID-19 pandemic has unveiled the harmful age-related deficit in the antiviral response in older people. Owing to this pandemic, numerous human clinical and epidemiological studies have been conducted, revealing that metformin as well as rapamycin significantly improve outcomes of COVID-19 patients with severe symptoms (Bischof et al., 2021; Hariyanto and Kurniawan, 2020). As a consequence, medical researchers worldwide now urge the testing of rapalogs, metformin and spermidine in large-scale clinical trials, to corroborate their promise of enhancing anti-pathogen immunity and boosting the immune system of elderly humans. Notably, the results of the Targeting Aging with Metformin (TAME) trial that enrolled 3000 participants (aged between 65 and 79) are expected to be a big leap forward (Justice et al., 2018; Kulkarni et al., 2020). We believe that conducting these clinical trials will pave the way towards the development of low-cost drugs that generally improve health of the elderly through modulating immune senescence and the biology of aging.
